# Accumulation of human full-length tau induces degradation of nicotinic acetylcholine receptor α4 *via* activating calpain-2

**DOI:** 10.1038/srep27283

**Published:** 2016-06-09

**Authors:** Yaling Yin, Yali Wang, Di Gao, Jinwang Ye, Xin Wang, Lin Fang, Dongqin Wu, Guilin Pi, Chengbiao Lu, Xin-Wen Zhou, Ying Yang, Jian-Zhi Wang

**Affiliations:** 1 Department of Pathophysiology, School of Basic Medicine and the Collaborative Innovation Center for Brain Science, Key Laboratory of Ministry of Education of China for Neurological Disorders, Tongji Medical College, Huazhong University of Science and Technology, Wuhan 430030, China; 2 Department of Physiology and Neurobiology, Henan province Key Laboratory of Brain Research, Xinxiang Medical University, Xinxiang 453003, China; 3 Co-innovation Center of Neuroregeneration, Nantong University, Nantong 226001, China

## Abstract

Cholinergic impairments and tau accumulation are hallmark pathologies in sporadic Alzheimer’s disease (AD), however, the intrinsic link between tau accumulation and cholinergic deficits is missing. Here, we found that overexpression of human wild-type full-length tau (termed hTau) induced a significant reduction of α4 subunit of nicotinic acetylcholine receptors (nAChRs) with an increased cleavage of the receptor producing a ~55kDa fragment in primary hippocampal neurons and in the rat brains, meanwhile, the α4 nAChR currents decreased. Further studies demonstrated that calpains, including calpain-1 and calpain-2, were remarkably activated with no change of caspase-3, while simultaneous suppression of calpain-2 by selective calpain-2 inhibitor but not calpain-1 attenuated the hTau-induced degradation of α4 nAChR. Finally, we demonstrated that hTau accumulation increased the basal intracellular calcium level in primary hippocampal neurons. We conclude that the hTau accumulation inhibits nAChRs α4 by activating calpain-2. To our best knowledge, this is the first evidence showing that the intracellular accumulation of tau causes cholinergic impairments.

Alzheimer’s disease (AD) is the most common neurodegenerative disease in the elderly. Pathologically, it is marked by the extracellular accumulation of plaques composed of β-amyloid peptide^1^ and intracellular neurofibrillary tangles that mainly contain the hyperphosphorylated tau proteins^2^. A massive loss of cholinergic neurons and nicotinic acetylcholine receptors (nAChRs) has been found in early stage of the disease onset^3^. The nAChRs interacts directly with β-amyloid and the cholinergic dysfunction in AD mouse model can be reversed by an anti-Aβ antibody^4^. Currently, the relationship of tau abnormality and cholinergic dysfunction/degeneration in the pathogenesis of AD is not understood.

The calpains are intracellular Ca^2+^-dependent cysteine proteases^5^. The two calpain subtypes are calpain-1 (μ-calpain) and calpain-2 (m-calpain) which differ in the calcium concentration required for their activation. Among different calpains, calpain-2 is particularly abundant in the central nervous system (CNS)^6^. In the AD brain there is an increased amount of calpain-2 co-located with neurofibrillary tangles^7^. Activation of calpain cleaves tau generating specific fragments (~35 kDa and ~17 kDa)^8^,^9^, which induce neuronal apoptosis in cerebellar granule cells^10^. Calpain cleaves many ion channels such as AMPAR and NMDAR subunits^11^,^12^. Activation of calpain mediates destabilization of AChR clusters at the neuromuscular junction^13^ and NMDA-induced excitotoxic impairment in the cholinergic nucleus basalis magnocellularis of Meynert^14^. Currently, it is not fully understood how calpain activation contributes to nAChRs degeneration in AD.

The nAChRs are the ligand-gated cation channel constituted of five subunits. In CNS, nAChRs regulate many pathophysiologic functions, such as anxiety, pain, and learning and memory^15^,^16^,^17^. The most abundantly expressed nAChR subunits in the CNS is α4, β2, and α7^18^, in which α4 subunit is markedly decreased in the hippocampus and temporal cortex of AD patients^19^. Cholinergic degeneration in AD is correlated with decline of the cognitive functions^20^. To explore the role of tau accumulation in cholinergic impairments, we overexpressed human full length tau (hTau) in cultured hippocampus neurons and in rat brain hippocampus, and measured the expression level of nAChR α4 and the function. We found that overexpression of hTau induced degradation of nAChR α4 with activation of calpains, and simultaneous inhibition of calpain-2 but not calpain-1 arrested the hTau-induced degradation of nAChR α4.

## Results

### Overexpression of hTau reduces protein level of α4 nAChR with an increased cleavage, but does not change the mRNA level of the receptor both *in vitro* and *in vivo*

**Figure 1:**
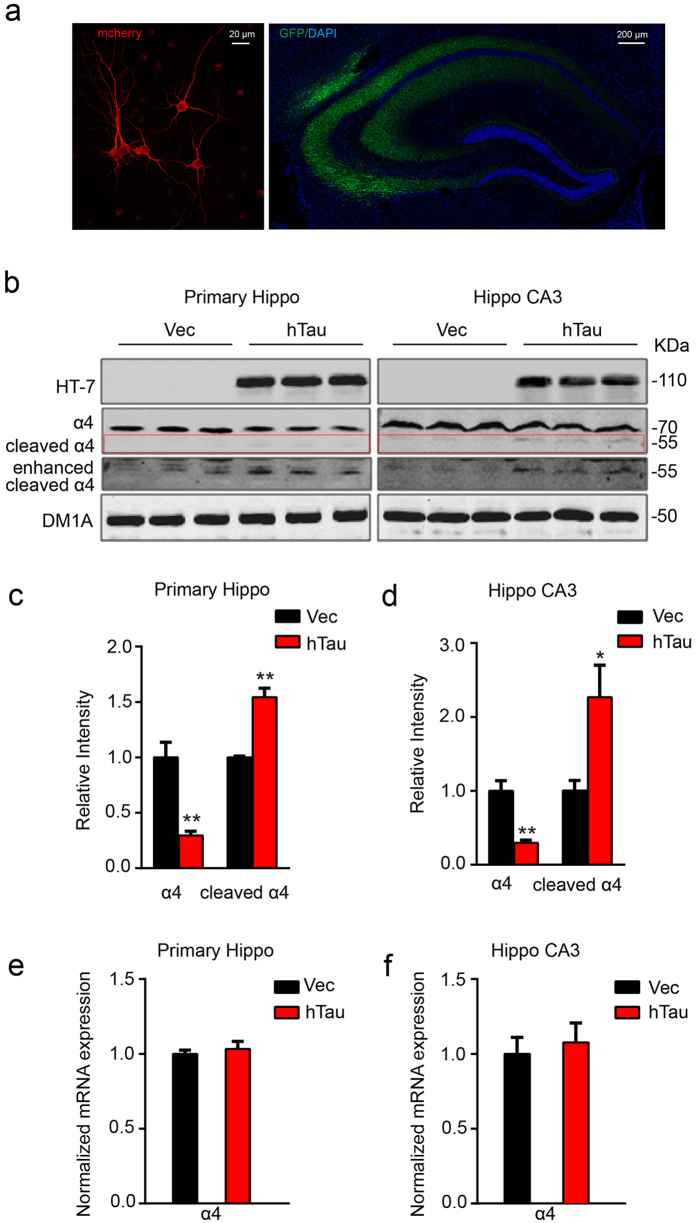
Overexpression of hTau reduces protein level of α4 nAChR with an increased cleavage of the receptor both *in vitro* and *in vivo*. (**a**) Representative images of the cultured hippocampus neurons, the lenti-mCherry-hTau or the vector was infected at 7 *DIV* and the neurons were cultured for another 5 day (left), or the one-month-old rat brain hippocampus after bilateral ventricular infusion of AAV-GFP-hTau (2 μl each side, 1.5 μl/min speed) at postnatal day 0–1 (P0-1) (right). (**b–d**) Western blotting data show that overexpression of hTau reduced α4 nAChR level in 12 *DIV* primary hippocampus neurons (left, from 3 independent cultures; two-sample unpaired t test, t_4_ = 4.949, *p* =  0.0078), or in rat hippocampal CA3 extracts (right, from at least 3 rats; two-sample unpaired t test, t_4_ = 5.862, *p* =  0.0042). The red dotted lines show the bands of cleaved α4 nAChRs fragment (two-sample unpaired t test, t_4_ = 6.634, *p* =  0.0027 in c; t_4_ = 3.982, *p* = 0.0164 in d). (**e,f**) Overexpressing hTau in primary neurons (two-sample unpaired t test, t_4_ = 0.5740, *p* =  0.5967) or rat hippocampus (two-sample unpaired t test, t_4_ = 0.4220, *p* =  0.6906) did not affect mRNA level of α4 nAChR measured by real-time fluorescent quantitative PCR. Data were expressed as mean ± SEM, *p < 0.05, **p < 0.01 *vs* control.

We first infected primary hippocampal neurons (7 *DIV*) with AAV-GFP-hTau or the vector as a control. After cultured for another 5 days, robust expression of the hTau was detected by fluorescence microscopy (Fig. 1a, left). Simultaneously, overexpression of hTau reduced the level of full-length α4 nAChR with an increased ~55kDa fragment when compared with the vector control (Fig. 1b,c). To further verify the effects of hTau on α4 nAChR *in vivo*, we infused AAV-GFP-hTau or the vector through cerebral ventricle bilaterally. After one month, robust expression of the hTau was detected in hippocampus, abundantly on the CA3 region (Fig. 1a, right). Therefore, we measured the α4 nAChR level by carefully dissecting CA3 subset. We observed that overexpression of hTau *in vivo* also reduced the protein level of α4 nAChR with an increased cleavage of the receptor (Fig. 1b,d). We also measured mRNA level of nAChR α4 by real-time fluorescent quantitative PCR, but no change was detected (Fig. 1e,f). These data suggest that overexpression of hTau reduces nAChR α4 protein level, and the mechanism may involve increased protein degradation.

### Overexpression of hTau diminishes α4 nAChR currents in hippocampal neurons

**Figure 2:**
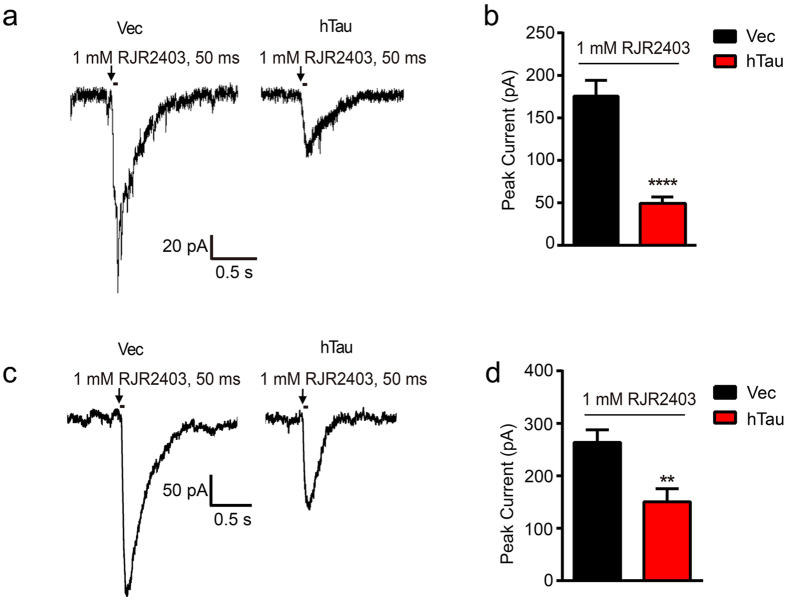
Overexpression of hTau diminishes α4 nAChR currents both in hippocampal CA3 and cultured hippocampal neurons. **(a)** The AAV-GFP-hTau or the vector was injected into the rat brain ventricle bilaterally (2 μl each side, 1.5 μl/min speed) at postnatal day 0–1 (P0-1), after one month, the electrophysiological recording was carried out on brain slices. The representative traces of RJR2403 (1 mM, 50 ms) show the activated whole-cell α4 nAChR currents in hippocampus CA3 pyramidal neurons. Arrows show the time points in which the drug was added. **(b)** Quantitative analysis data show mean α4 nAChRs current peak values in neurons with overexpression of hTau (n = 9 slices, 3 mice) or the vector (n = 7 slices, 3 mice) (two-sample unpaired t test, t_14_ = 7.080, *p *< 0.0001). **(c)** The cultured hippocampus neurons (7 *DIV*) were infected with lenti-mCherry-hTau or the vector for 5 days. The α4 nAChR currents induced by RJR2403 (1 mM, 50 ms) were recorded by whole-cell patch clamp and the representative traces were shown (n = 7 neurons per group). **(d)** Quantitative analyses were performed (two-sample unpaired t test, t_12_ = 3.266, *p* = 0.0068). Data were expressed as mean ± SEM, **p < 0.01, ****p < 0.0001 *vs* vector group.

In the CNS, α4 subunits are major components of nAChRs which are unselective cation channels. To further explore the influence of hTau on the function of the channel, we infected the rat by intracerebroventricular infusion of AAV-GFP-hTau or the vector at postnatal 0–1 day (P0-1), after 3 weeks, whole-cell electrophysiological recordings were performed on the brain slices. A selective nAChR α4 agonist RJR2403 (1 mM, duration 50 ms) in the injection pipette was applied to induce α4 nAChR current (Fig. 2a). The α4 nAChR responses recorded from control brain slices showed a mean current amplitude of 178.70 ± 21.64 pA (n = 7 slices from 3 mice) elicited by 1 mM RJR2403, compared with the mean current amplitude of 47.33 ± 10.46 pA (n = 9 slices from 3 mice) in brain slices overexpressed with hTau (Fig. 2b). In primary hippocampal neurons, it was also observed that overexpression of hTau weakened α4 nAChR currents induced by RJR2403 (Fig. 2c,d). No differences in current duration were found between the vector and hTau groups. These data suggest that overexpression of hTau in the hippocampus CA3 pyramidal neurons significantly inhibits the α4 nAChR peak currents both *in vitro* and *in vivo*.

### Overexpression of hTau activates calpains with no effect on caspase-3

**Figure 3:**
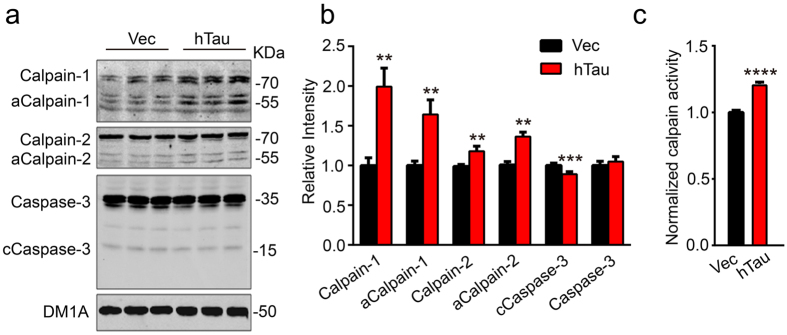
Overexpression of hTau activates calpains. The 7 *DIV* hippocampal neurons were infected with lenti-mCherry-hTau virus or the vector for 5 days, then the cell extracts were prepared for Western blotting **(a,b)** or the calpain activity assay. **(c)** Overexpression of hTau increased total levels of calpain-1 (two-sample unpaired t test, t_4_ = 3.933, *p* = 0.0043), calpain-2 (two-sample unpaired t test, t_4_ = 4.903, *p* = 0.0017), the activated calpain-1 (aCalp-1) (two-sample unpaired t test, t_4_ = 3.724, *p* = 0.0074), the activated calpain-2 (aCalp-2) (two-sample unpaired t test, t_4 _= 5.143, *p* = 0.0068), and the calpain activity (two-sample unpaired t test, t_10 _= 9.639, *p* < 0.0001) with a decreased level of cleaved caspase-3 (two-sample unpaired t test, t_4_ = 5.456, *p* = 0.0003). The data were from at least three independent experiments and were expressed as mean ± SEM, **p < 0.01, ***p < 0.001, ****p < 0.0001 *vs* vector.

To explore the mechanisms underlying the degradation of α4 nAChR by overexpression of hTau, the proteolysis system including calpains and caspase-3 was measured by Western blotting. We observed that the levels of total calpain-1, total calpain-2, activated calpain-1 and calpain-2 (~58 kDa) were significantly increased in primary hippocampal neurons with overexpression of hTau (Fig. 3a,b). To further confirm the results, we measured the activity of calpains by activity assay and an accorded activation of calpain was also detected in primary hippocampus neurons after overexpression of hTau (Fig. 3c). We also measured the cleavage of capspase-3 by Western blotting. We found that the cleavage (~17 kDa fragment, active form) of caspase-3 (35 kDa) was decreased in the neurons with overexpression of hTau (Fig. 3a,b), which coincidentally agree with our previous findings^21^,^22^. These data demonstrate that overexpression of hTau activates calpains with inhibition of caspase-3.

### Inhibition of calpain-2 but not calpain-1 attenuates the hTau-induced cleavage of α4 nAChR in hippocampus neurons

**Figure 4:**
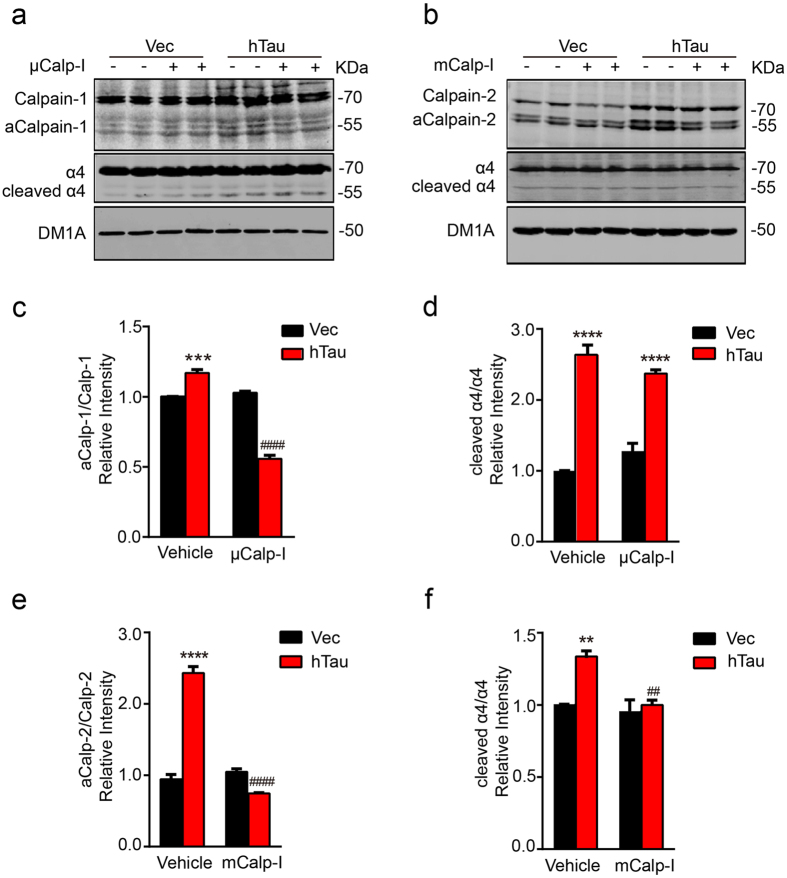
Inhibition of calpain-2 but not calpain-1 attenuates the hTau-induced cleavage of α4 nAChR in hippocampus neurons. **(a,b)** The 7 *DIV* hippocampal neurons were infected with lenti-mCherry-hTau virus or the vector. At 10 *DIV*, the neurons were treated with 2 μM μ-calpain inhibitor PD151746 (μCalp-I) or 10 μM m-calpain inhibitor (mCalp-I) or the vehicle for 48 h, and then the cell lysates were prepared for Western blotting. **(c–f)** Quantitative analyses showed that application of μCalp-I decreased aCalp-1/Calp-1 ratio (two-way ANOVA, factor infection: *F*_1,8_ = 69.642, *p *< 0.0001; factor drug: *F*_1,8_ = 258.146, *p *< 0.0001; infection × drug: *F*_1,8_ = 307.814, *p *< 0.0001; Tukey’s *post hoc* test), inhibited calpain-1 activation, but did not block α4 degradation induced by calpain-1 activation (two-way ANOVA, factor infection: *F*_1,8_ = 223.775, *p *< 0.0001; factor drug: *F*_1,8_ = 0.003, *p*  = 0.957; infection × drug: *F*_1,8_ = 8.841, *p* = 0.018; Tukey’s *post hoc* test). Application of mCalp-I inhibited calpain-2 (two-way ANOVA, factor infection: *F*_1,8_ =  94.811, *p *< 0.0001; factor drug: *F*_1,8_ = 193.761, *p* < 0.0001; infection × drug: *F*_1,8_ = 203.197, *p* < 0.0001; Tukey’s *post hoc* test) and attenuated α4 degradation (two-way ANOVA, factor infection: *F*_1,8_ = 25.679, *p* = 0.0010; factor drug: *F*_1,8_ = 20.462, *p* = 0.0019; infection × drug: *F*_1,8_ = 14.5184, *p* = 0.0052; Tukey’s *post hoc* test). The data were from at least three independent experiments and expressed as mean ± SEM, **p < 0.01, ***p < 0.001, ****p < 0.0001 *vs* Vec+Vehicle; ^##^*p *< 0.01, ####*p* < 0.0001 *vs* hTau+Vehicle.

To confirm the role of calpains in hTau-induced cleavage of α4 nAChR, we used inhibitors of calpains. The hippocampus neurons were treated with calpain-1 inhibitor PD151746 (2 μM, μCalp-I) or calpain-2 inhibitor (10 μM, mCalp-I) for 48 h, and then the level of α4 nAChR was detected by Western blotting. We found that the application of μCalp-I to primary hippocampus neurons inhibited activation of calpain-1, displayed by the weaker staining of the activated calpain-1 (~58 kDa) (Fig. 4a,c), however, inhibition of calpain-1 did not relieve the hTau-induced cleavage (~55 kDa) of α4 nAChR (Fig. 4a,d). On the other hand, application of mCalp-I to primary hippocampus neurons inhibited the activity of calpain-2 with a remarkable reduction of the cleavage α4 nAChR (~55 kDa) (Fig. 4b,e,f). These results indicate that inhibition of calpain-2 but not calpain-1 attenuates the cleavage of α4 nAChR induced by overexpression of hTau in hippocampus neurons.

### Inhibition of calpain-2 restores the hTau-induced inhibition of α4 nAChR currents in hippocampus neurons

**Figure 5:**
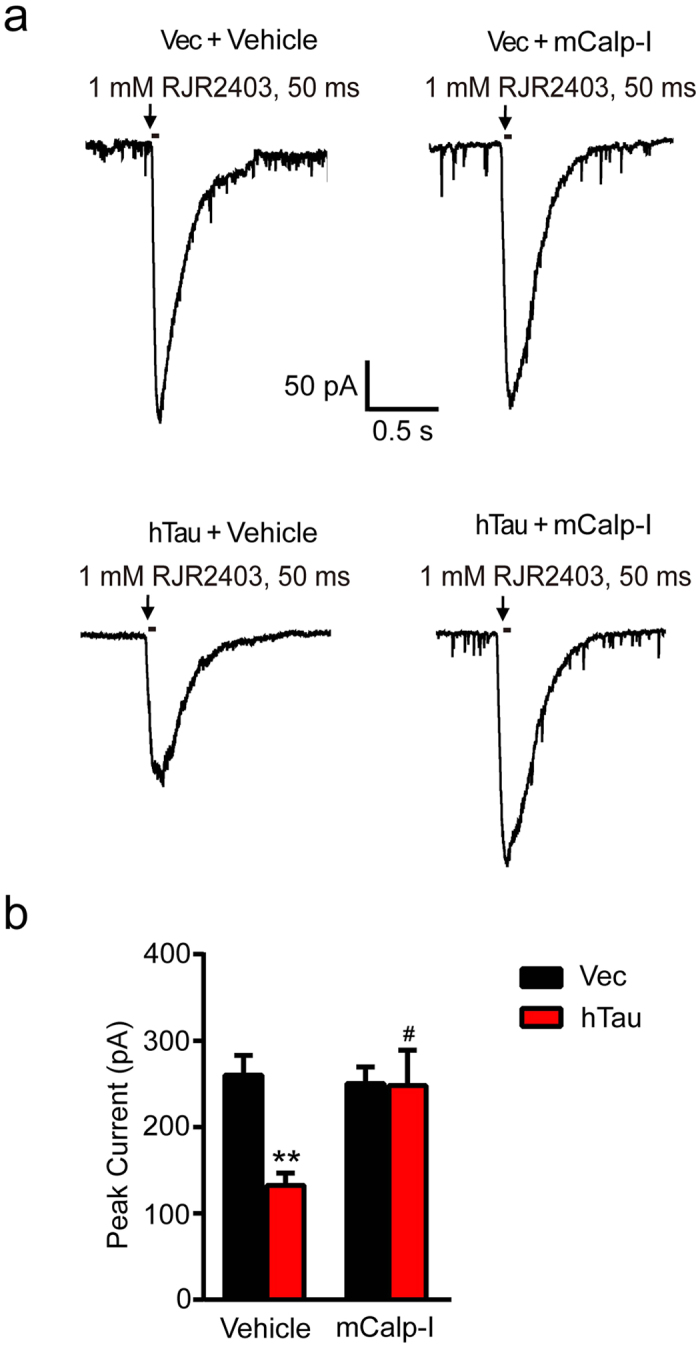
Inhibition of calpain-2 rescues the hTau-induced inhibition of α4 nAChR currents in hippocampal neurons. (**a**) The cultured hippocampal neurons (7 *DIV*) were infected by lenti-mCherry-hTau virus or the vector. After 3 days, the neurons were treated with 10 μM mCalp-I for 48 h, and then the α4 nAChR agonist RJR2403 (1 mM, 50 ms) was puffed to elicit α4 current recorded by whole-cell patch clamp. (**b**) The amplitude of currents were analyzed (two-way ANOVA, factor infection: *F*_1,24_ = 6.492, *p* = 0.0177; factor drug: *F*_1,24_ = 4.380, *p* = 0.0471; infection × drug: *F*_1,24_ = 6.097, *p* = 0.0210; Tukey’s *post hoc* test). At least 7 neurons from each group were recorded and the data were expressed as mean ± SEM. **p < 0.01 *vs* Vec+Vehicle; ^#^*p *< 0.05, *vs* hTau+Vehicle.

By using electrophysiological recording, we further studied the effect of calpain-2 inhibitor on α4 nAChR currents in the cultured hippocampal neurons with overexpression of vector or hTau. As shown in Fig. 2, overexpression of hTau significantly reduced the nAChR currents compared with the vector group (Fig. 5a,b), and simultaneous inhibition of calpain-2 restored the hTau-induced inhibition of the α4 nAChR currents with no significant effect on the vector-expressing group (Fig. 5a,b).

### Overexpression hTau in hippocampal neurons increases the level of intracellular basal calcium

**Figure 6:**
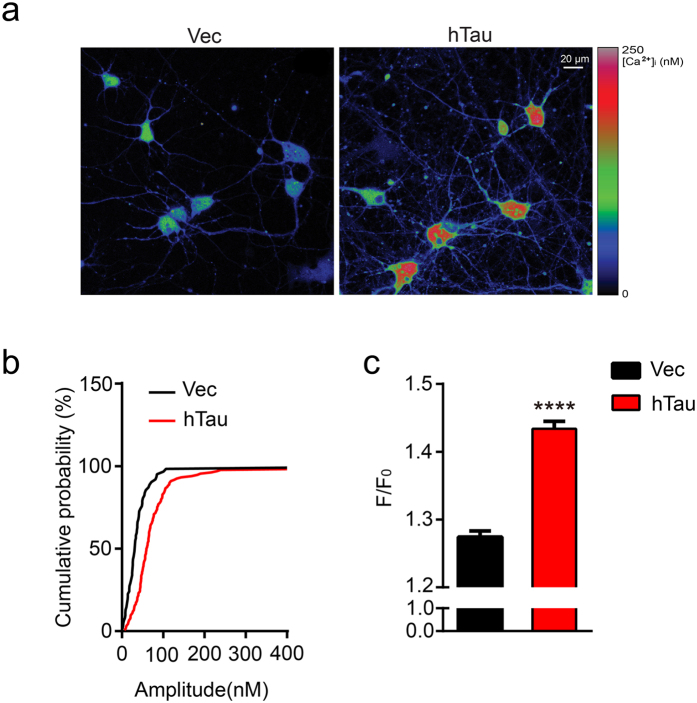
Overexpression hTau in hippocampal neurons increases the level of intracellular basal calcium. The 7 *DIV* hippocampal neurons were infected with lenti-mCherry-hTau virus or the vector for 5 days, and then the intracellular basal calcium level was measured by Fluo-3 AM expressed as F/F_0_. **(a)** The representative imaging in primary hippocampus neurons overexpressing with hTau and the control virus. **(b)** Cumulative distribution of basal [Ca^2+^]_i_ showed higher basal calcium level in hippocampal neurons expressing hTau (n = 112) than in neurons expressing vector (n = 62). **(c)** The fluorescence intensity of intracellular basal calcium was quantitatively analyzed (two-sample unpaired t test, t_172_ = 9.232, *p *< 0.0001), F is the real time fluorescence intensity of Fluo-3 AM, F_0_ is the mean fluorescence intensity of the baseline recorded. The values of calcium signal were expressed by F/F_0_. Data were expressed as mean ± SEM. ****p < 0.0001 *vs* vector group.

Calpains are cellular calcium-dependent proteases. To explore the mechanism underlying the hTau-induced calpain activation, we measured the intracellular calcium by Fluo-3 AM imaging (Fig. 6a). Cumulative distribution of basal [Ca^2+^]_i_ is used to show the difference of basal calcium level between hippocampal neurons overexpressing hTau or the control virus (Fig. 6b). The neurons with hTau overexpression showed a mean basal [Ca^2+^]_i_ of 184.82 ± 20.63 nM (n = 112 cells) compared with the mean basal [Ca^2+^]_i_ of 92.3 ± 19.80 nM (n = 62 cells) in the control neurons (Fig. 6c). These data suggest that overexpression of hTau in primary hippocampus neurons significantly increased basal [Ca^2+^]_i_ that can activate calpains.

## Discussion

AD is the most common neurodegenerative disease characterize by loss of cholinergic neurons, which begins in entorhinal cortex at early stage of the disease onset and later spreads to the limbic hippocampus and neocortex with reduced nAChR^23^,^24^. Coincidentally, tau abnormality also follows the same brain regions with disease progression^25^. Previous studies show that the α4 nAChR subunit was exclusively expressed in neurons, whereas α7 nAChR subunit was strongly expressed in neurons and astrocytes. The numbers of α4- and α7-positive neurons in the hippocampus and temporal cortex of AD patients carrying Swedish APP 670/671 mutation were significantly decreased compared with the sporadic AD patients^26^. Laboratory studies show that application of α7 nAChR agonist or antagonist attenuates the Aβ-induced tau hyperphosphorylation^27^, and activation of α7 decreases tau phosphorylation by inhibiting GSK-3β^28^. These data suggest that α4 or α7 nAChR can be upstream of tau phosphorylation, though complicated results have received. Another laboratory study demonstrated that extracellular application of tau proteins provoked an increase of the intracellular calcium which may involve muscarinic receptors^29^. To date, it has not been reported whether and how intracellular tau accumulation as seen in the AD brains may affect cholinergic functions. By overexpressing human full length tau in hippocampal neurons, we found here that intracellular accumulation of hTau significantly reduced nAChR α4 subunit, and the mechanisms involve activation of the calcium-dependent calpain-2. These data reveal the mechanisms underlying the hTau-induced cholinergic impairment, suggesting that targeting calpain-2 may be potential for arresting the hTau-induced toxicities.

In the AD brains, neurons heavily labeled with the hyperphosphorylated tau had low mRNA level of α4 and α7^30^, and a significant reduction of α4 but not α3 or α7 protein was also detected^19^,^31^. In APP or APP/PS-1transgenic mice, level of α4 subunit was unchanged^32^,^33^. These data suggest that the influence of Aβ on nAChR α4 may be less than that of tau, which is supported by our current findings. Overexpression of hTau did not change the mRNA level of nAChR α4, suggesting that the hTau may affect protein degradation but not the transcription of nAChR α4.

The nAChRs are ligand-gated cation channels which are opened in response to the binding of acetylcholine, nicotine or specific agonist^34^. It mediates fast synaptic transmission and participates in synaptic plasticity and memory formation^35^. Using whole-cell electrophysiological recordings, we also found that overexpression of hTau significantly attenuated α4 nAChR peak current provoked by a selective nAChR α4 agonist RJR2403. This functional change of the α4 nAChR was in accord with the protein expression of α4 nAChR.

Calpains are calcium-dependent proteases, which are regulated by intracellular [Ca^2+^]_i_^5^. Based on the calcium concentration dependence, two types of calpains, namely μ-calpain (or calpain-1) and m-calpain (or calpain-2), have been identified in central nervous system^36^. In the AD brains, the activity of calpain-1 shows seven-folds higher than that of the age-matched controls^37^, while the activated calpain-2 was co-localized with 50–75% of neurofibrillary tangles^38^. NMDA receptor mediates tau-induced neurotoxicity by activating calpain^39^. We found here that tau accumulation was accompanied by activation of both calpain-1 and 2, and only simultaneous inhibition of calpain-2 but not calpain-1 attenuated the hTau-induced cleavage of nAChR α4 subunit. This finding indicates that calpain-2 plays a crucial role in mediating the hTau-induced α4 nAChR degradation, which not only present direct evidence to link tau abnormality to cholinergic impairments in AD, but also provide potential drug target (calpain-2) for tau-related treatment. Indeed, calpain inhibitor can reduce tau hyperphosphorylation and Aβ production with attenuation of synapse pathologies and memory deficits in mice^40^,^41^. The phosphorylated fetal tau isoforms are readily proteolyzed by calpain-2, while the PHF-tau was more resistant to the proteolysis by calpain^42^. Additionally, we reported that overexpressing hTau inhibited caspase-3 and cell apoptosis^43^,^44^ while proteasome inhibition induces tau accumulation^45^,^46^. Here, we also observed that overexpression of hTau in primary neurons decreased the cleavage of caspase-3, which nicely reproduced our previous observation.

Calpain activity is regulated mainly by intracellular calcium^47^, and deregulation of calcium homeostasis has occurred in early stage of AD^48^. Aβ can increase intracellular calcium^49^, whereas expression of P301L mutated 0N4R-tau in rTg4510 model did not disrupt intracellular calcium homeostasis^50^. We found here that overexpression of hTau (2N4R) significantly increased the intracellular basal calcium level. The discrepancy may result from different tau isoforms applied, i.e., wild type *vs* mutant and 0N4R *vs* 2N4R, and the full length 2N4R tau containing 441 amino acid residues used in our present study seems most cytotoxic^51^,^52^. Interestingly, the increased level of intracellular calcium induced by overexpression of hTau seems not enough to activate m-calpain. In the AD brains, the decrease of nAChRα4 subunit at protein level was correlated with an increased lipid peroxidation^53^. As phospholipids, such as phosphatidylinositol 4,5-bisphosphate, can lower the Ca^2+^ concentration required for autolysis of either calpain-1 or calpain-2 by three- to six-fold^54^, we speculate that overexpression of hTau may also affect phospholipid metabolism, which deserves further investigation. A previous study showed that extracellular treatment of cells with tau also increased Ca^2+^ concentration^29^. Although both extracellular and intracellular tau caused calcium increase, the cellular and molecular mechanism may be different, which deserves further investigation.

Taken together, we find in the present study that overexpression of hTau increases intracellular calcium, which in turn activates calpain-2 and induces degradation of α4 nAChR.

## Methods

### Virus construction, antibodies and chemicals

The plasmid pEGFP-hTau-2N4R encoding human full-length microtubule-associated protein tau (hTau) was a generous gift from Dr. Fei Liu (Jiangsu Key Laboratory of Neuroregeneration). Based on that, lenti-CMV-hTau-mCherry and AAV-CMV-hTau-GFP were reconstructed and packaged in our laboratory. Multiplicity of infection (MOI) of 10 was used to virus infection *in vitro* and *in vivo*.

Rabbit monoclonal antibodies against Nicotinic Acetylcholine Receptor (nAChR) α4, calpain-1, and calpain-2 were from Abcam (USA). Rabbit polyclonal antibodies caspase-3 and the cleaved caspase-3 were obtained from Cell Signaling. Mouse monoclonal antibody HT-7 against human tau and DM1A against tubulin was from Thermo Fisher (USA) and Sigma-Aldrich (USA), respectively.

RJR2403 (a selective nAChR α4 agonist) was from Tocris(UK). Fluo3-AM was from Dojindo (Japan). The μ-calpain inhibitor 3-(5-fluoro-3-indolyl)-2-mercapto-(Z)-2-propenoic acid (μCalp-I, also known as PD151746) and m-calpain inhibitor (mCalp-I, also known as calpain inhibitor IV) were from EMD Millipore (Germany). Calpain activity assay kit was from Abcam (USA). All other reagents were obtained from Sigma-Aldrich (USA).

### Primary hippocampal neuronal cultures

Primary hippocampus neurons were prepared from 17 to 18-day-old rat embryos. Hippocampus were dissected and gently minced in Hank’s buffered saline solution (HBSS), then suspended in 0.25% trypsin solution at 37 °C for 15 min. Neurons were plated in culture dishes coated with 100 ug/ml poly-L-lysine and cultured *in vitro* in neurobasal medium supplemented with 2% B-27 and 1 × GlutaMAX. Neurons were infected with lenti-mCherry and lenti-mCherry-hTau at 7 days *in vitro* (*DIV*). Cultured 12 *DIV* hippocampal neurons were used for following experiments. All cell culture reagents were purchased from Thermo Fisher Scientific Inc.

### Animals and stereotaxic injection

Postnatal day 0–1 (P0-1) Sprague-Dawley rats and their female parents were supplied by the Experimental Animal Central of Tongji Medical College. All animal experiments were performed according to the Policies on the Use of Animals and Humans in Neuroscience Research revised and approved by ethical committee of Tongji Medical College. Rats were housed with free access to food and water under 12h/12h light-dark cycle. The P0-1 pups were anesthetized for 5 min by being wrapped with ice pack. When all pedal reflexes were abolished, it was placed on a square glass in a prone position. At least 10^12 ^U/ml AAV-GFP-hTau or control virus was injected bilaterally into lateral ventricle (2 μl each side, 1.5 μl/min speed) by a syringe (10 μl, 30 gauge 1/2 inch, Hamilton,803803) at the coordinates of 0.5 mm anterior to posterior bregma (AP), 1.0 mm mid to lateral (ML), 2.5 mm dorsal to ventral (DV). The injection needle was kept *in situ* for 2 min following virus infusion to avoid spread of the virus solution along the track. The pups were immediately put on the heating palm to make them come around after injection, and for further recovery they were returned to their female parents. All surgical procedures were completed under sterile conditions. The pups were fed by their female parents up to P21–30.

### Electrophysiological recordings

Postnatal day 21–30 SD rats of either sex were used for all our electrophysiology experiments. Rats were deeply anaesthetized by intraperitoneal injection of chloral hydrate (300 mg/kg, Sigma-Aldrich). When all pedal reflexes were abolished, brains were removed and placed in ice-cold oxygenated slicing solution containing the following (in mM): 225 sucrose, 3 KCl, 1.25 NaH_2_PO_4_, 24 NaHCO_3_, 6 MgSO_4_, 0.5 CaCl_2_ and 10 D-glucose. Coronal slices (350 μm thick) containing the dorsal hippocampus were cut at 4–5 °C in the slicing solution using a Leica VT1000S vibratome (Leica, Germany), and then transferred to an incubation chamber filled with oxygenated slicing solution in a 30 °C water bath for 1 hour before being recorded. For primary cultured hippocampal neurons, neurons at 7 *DIV* were infected with lenti-CMV-hTau-mCherry and the vector virus, three days later, treated with m-calpain inhibitor (mCalp-I, 10 μM) for 48 h.

Whole-cell recordings were performed using a patch clamp amplifier (Multiclamp700B, Axon Ins.) and an upright infrared-DIC microscopy with a 40×water objective at 31 ± 1 °C by an automatic temperature controller (Warner Ins.). During recordings, slices were perfused continuously at 2 ml/min with extracellular solution containing (in mM): 146 NaCl; 10 Hepes; 2.5 KCl; 2 CaCl_2_; 2 MgCl_2_; 5 glucose (pH 7.3), aerated with 95% O_2_ and 5% CO_2_ during incubation and recording. Neurons of hippocampus CA3 region or primary culture were visualized for whole-cell recordings. The whole-cell recording pipette has tip resistance (2–5 MΩ), was filled with a solution containing (in mM): 140 potassium gluconate, 5 KCl, 10 HEPES, 0.2 EGTA, 2 MgCl_2_, 4 MgATP, 0.3 Na_2_GTP and 10 Na_2_-phosphocreatine at pH 7.2. Data acquisition was performed using a digitizer (DigiData 1440, Axon Ins.) and off-line analysis was carried out with pClamp 10.0 (Axon Ins.). The whole-cell currents were filtered at 2 kHz and sampled at 10 kHz. Series resistance (<20 MΩ) or membrane resistance (300–500 MΩ) was monitored throughout the whole-cell recording and data were discarded if the resistance changed by more than 20%.

For recording currents, the membrane potential was holding at -60 mV at voltage-clamp model. RJR2403 (1 mM) was puffed by pressure from a micropipette using a picospritzer III (World Precision Instruments, Stevenage, Hertfordshire, UK) every 30–60 s, at 10 psi for a duration of 50 ms from patch pipettes of the same dimensions as those used for recording (tip 2–4 μm), with the pipette tip locating at 20–40 μm away from the soma surface, so that the cell recorded could be totally immersed into the drug solution during perfusion^55^. The peak amplitude of the current response was measured and calculated.

### Calcium imaging

For calcium imaging, the primary hippocampal neurons were plated in the coverslip (diameter  = 30 mm) coated by with 100 μg/ml poly-D-lysine and cultured for 7 days, were transfected with lenti-mCherry and lenti-mCherry-hTau. The 12 *DIV* hippocampal neurons were loaded with the fluorescent calcium dye Fluo3-AM (5 μM, Dojindo, Japan) for 30 min at room temperature in Krebs-HEPES buffer containing (in mM): 135 NaCl, 6 KCl, 2 CaCl_2_, 1.2 MgCl_2_, 10 D-glucose, 10 HEPES at pH 7.4. The coverslips were washed and cells were allowed to deesterify for at least 20 min in indicator-free Krebs-HEPES buffer before imaging. The coverslips were mounted in a perfusion chamber, fluorescent signals in cells were recorded by time-series scan imaging on confocal microscope (Zeiss LSM 510, 40×, 1.3 NA objectives) equipped with an argon laser (488 nm). Two thirds of cell somas were set as the region of interest (ROI) for image analysis. The calcium signal was self-normalized by the real-time recorded values (F) and the mean baseline values recorded at the first 3 min (F_0_) and expressed as F/ F_0_^56^. Calcium imaging was performed at room temperature and the sampling rate was 1 Hz. To determinate [Ca^2+^]_i_, high calcium solution (5 mM Ca^2+^) and Ca^2+^-free/EGTA solution were perfused respectively. The fluorescence was acquired as F_max_ and F_min_. For the recorded fluorescence F, [Ca^2+^]_i_ = [(F-Fmin)/(Fmax-F)] × K_d_, (K_d_ = 400 nm)^57^. The quantitative scale was received by using the analysis software (ZEN2009) affiliated with the two photon microscope (Zeiss LSM 510, 40×, 1.3 NA objectives).

### Western blotting

After lentivirus infection for 5 days, 12 *DIV* primary hippocampal neurons were collected and homogenized in RIPA buffer supplemented with 0.1% cocktail and 1 mM PMSF for whole cell lysates at 4 °C. For rats experiments, the CA3 regions were isolated carefully with a sharp needle-tip^58^. Then the protein lysates were boiled at 95 °C for 5 min in the loading buffer (50 mM Tris-HCl, pH 7.6, 2% SDS, 10% glycerol, 10 mM DTT, and 0.2% bromphenol blue), were separated by 10% SDS-polyacrylamide gel electrophoresis (SDS-PAGE) and transferred onto 0.45 μm nitrocellulose membranes (Whatman). The membranes were blocked in 5% nonfat milk dissolved in TBST (50 mM Tris-HCl, pH 7.6, 150 mM NaCl, 0.2% Tween-20) for 1 h and incubated with primary antibodies against nAChR α4 (1:1000, Abcam, ab124832), calpain-1(1:500, Abcam, ab39170), calpain-2 (1:500, Abcam, ab39165), caspase-3(1:1000, Cell Signaling, 9662), cleaved caspase-3(1:1000, Cell Signaling, 9661), HT-7(1:1000, Thermo Fisher, MN1000), DM1A (1:1000, Sigma, T9026) at 4 °C overnight. Membranes were then incubated with a secondary antibody (1:10000, Odessey) at room temperature. Immunoreactive bands were visualized with Odyssey Infrared Imaging System (Li-cor Biosciences, Lincoln, NE) and quantified by Image J software. The results from three independent experiments were analyzed. The full Western blottings are shown as Supplementary Fig. S1–3.

### Quantitative RT-PCR analysis

Total RNA was extracted from cell and tissue samples using TRIzol reagent (Invitrogen) according to the manufacturer’s protocol. RNA_260/280_ was measured spectrophotometrically for determining the concentration and purity. 1 g RNA was reverse transcribed into single-strand cDNA using ReverTra Ace-α-cDNA Synthesis Kit (TOYOBO). Quantitative PCR amplification was performed using CFX96 Real-Time PCR Detection System (Bio-Rad) and SYBR Green Premix Ex TaqTM (TaKaRa). The reaction conditions for PCR is an initial denaturation at 95 °C for 3 min, followed by 40 cycles of 95 °C for 10 s, 60 °C for 30 s, and extension at 72 °C for 30 s. Primers (GeneCopoeia) for qPCR analysis of nicotinic α4 nAChR and GAPDH were designed and synthesized. The comparative cycle threshold (CT) method was used to quantify the abundance of α4 nAChR transcripts.

### Calpain activity assay

7 *DIV* primary hippocampal neurons were infected with lenti-mCherry-hTau and the control virus. After 5 days, cell lysates were prepared by the extraction buffer provided with the kit. 100 μg lysates were incubated with 5 μL of calpain substrate Ac-LLY-amino-4-trifluoromethylcoumarin(Ac-LLY-AFC) at 37 °C for 60 minutes protected from light. Calpain activity was detected by measuring the fluorescence of free AFC released from the substrate at excitation/emission (Ex/Em) wavelengths of 400/505 nm on a fluorescence plate reader (BioTek, USA) according to the manufacturer’s instruction.

### Statistical Analysis

Data were expressed as means ± SEM, and analyzed using Graphpad prism 6.0 statistical software. Statistical analysis were performed using two-sample unpaired t test for pairwise comparisons and two-way ANOVA with Tukey’s post-hoc corrections for multiple comparisons. The test with *p *< 0.05 was considered as statistically significant.

## Additional Information

**How to cite this article**: Yin, Y. *et al*. Accumulation of human full-length tau induces degradation of nicotinic acetylcholine receptor α4 *via* activating calpain-2. *Sci. Rep*. **6**, 27283; doi: 10.1038/srep27283 (2016).

## Supplementary Material

Supplementary Information
