# Correction: Accumulation of human full-length tau induces degradation of nicotinic acetylcholine receptor α4 *via* activating calpain-2

**DOI:** 10.1038/s41598-026-48543-x

**Published:** 2026-05-19

**Authors:** Yaling Yin, Yali Wang, Di Gao, Jinwang Ye, Xin Wang, Lin Fang, Dongqin Wu, Guilin Pi, Chengbiao Lu, Xin-Wen Zhou, Ying Yang, Jian-Zhi Wang

**Affiliations:** 1 Department of Pathophysiology, School of Basic Medicine and the Collaborative Innovation Center for Brain Science, Key Laboratory of Ministry of Education of China for Neurological Disorders, Tongji Medical College, Huazhong University of Science and Technology, Wuhan, 430030 China; 2 Department of Physiology and Neurobiology, Henan province Key Laboratory of Brain Research, Xinxiang Medical University, Xinxiang, 453003 China; 3 Co-innovation Center of Neuroregeneration, Nantong University, Nantong, 226001 China

Correction to: *Scientific Reports* 10.1038/srep27283, published online 09 June 2016

This Article contains errors.

 In Figure 1b, the cropped blots of the loading control, DM1A, are incorrect. The correct Figure 1 and its accompanying legend appear below. The original blots corresponding to the correct DM1A cropped blots are included in the revised Supplementary Information file linked to this notice.

**Fig. 1:**
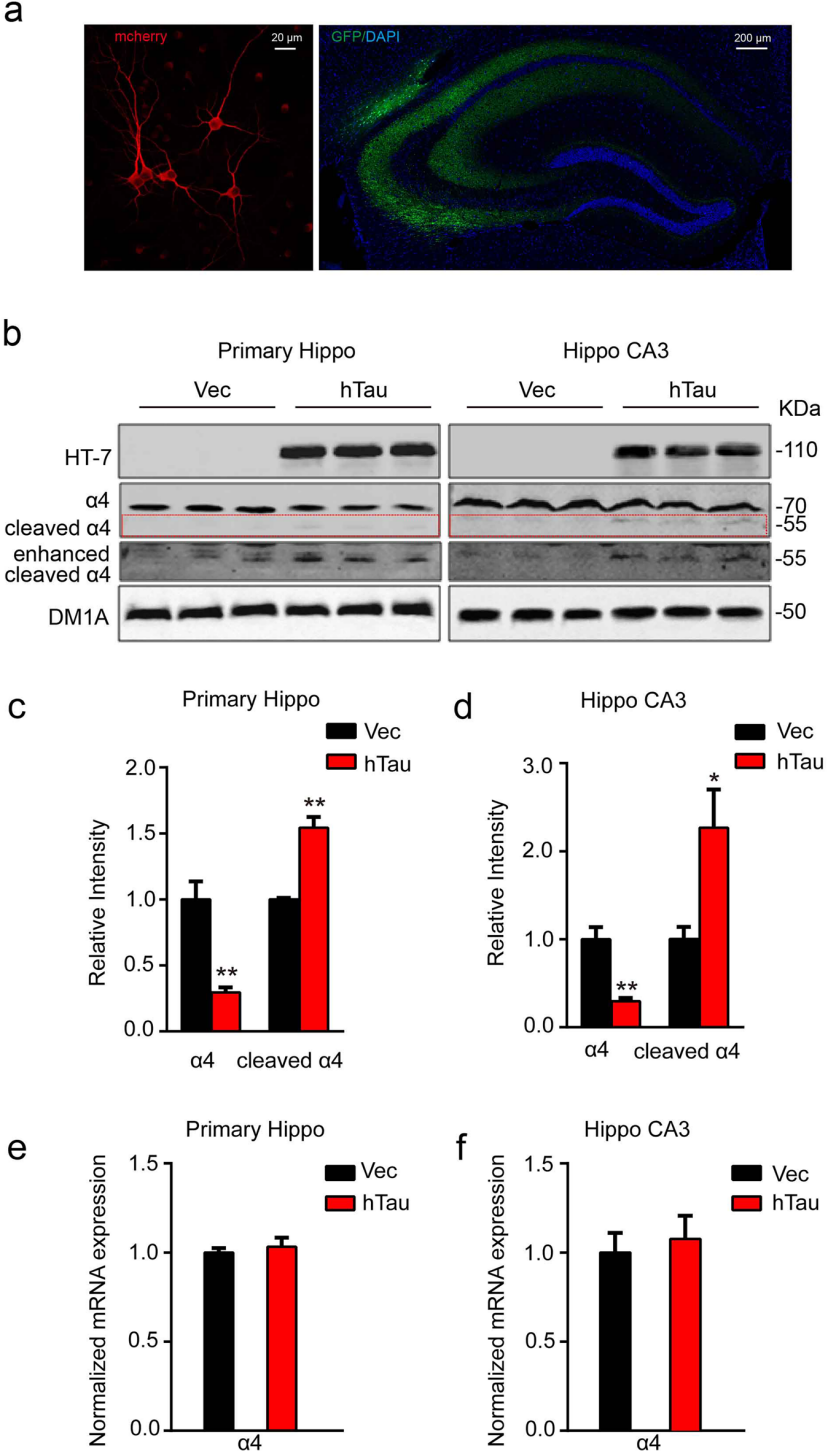
Overexpression of hTau reduces protein level of α4 nAChR with an increased cleavage of the receptor both *in vitro* and *in vivo*. (**a**) Representative images of the cultured hippocampus neurons, the lenti-mCherry-hTau or the vector was infected at 7 *DIV* and the neurons were cultured for another 5 day (left), or the one-month-old rat brain hippocampus after bilateral ventricular infusion of AAV-GFP-hTau (2 μl each side, 1.5 μl/min speed) at postnatal day 0–1 (P0-1) (right). (**b–d**) Western blotting data show that overexpression of hTau reduced α4 nAChR level in 12 *DIV* primary hippocampus neurons (left, from 3 independent cultures; two-sample unpaired t test, t_4_ = 4.949, *p* = 0.0078), or in rat hippocampal CA3 extracts (right, from at least 3 rats; two-sample unpaired t test, t_4_ = 5.862, *p* = 0.0042). The red dotted lines show the bands of cleaved α4 nAChRs fragment (two-sample unpaired t test, t_4_ = 6.634, *p* = 0.0027 in c; t_4_ = 3.982, *p* = 0.0164 in d). (**e,f**) Overexpressing hTau in primary neurons (two-sample unpaired t test, t_4_ = 0.5740, *p* = 0.5967) or rat hippocampus (two-sample unpaired t test, t_4_ = 0.4220, *p* = 0.6906) did not affect mRNA level of α4 nAChR measured by real-time fluorescent quantitative PCR. Data were expressed as mean ± SEM, *p < 0.05, **p < 0.01 *vs* control.

These changes do not affect the conclusions of the Article

## Supplementary Information

Supplementary Information.

