# Supplementary material for: Correction: Accumulation of human full-length tau induces degradation of nicotinic acetylcholine receptor α4 via activating calpain-2
Source: Sci Rep. 2026 May 19;16:15498. doi: 10.1038/s41598-026-48543-x (PMC13187463; doi:10.1038/s41598-026-48543-x)
Supplement: Supplementary file 1 — Supplementary Information. [file 41598_2026_48543_MOESM1_ESM.pdf]

Supplementary information

Accumulation of human full-length tau induces degradation of nicotinic acetylcholine receptor α4 *via* activating calpain-2

Yaling Yin[1], Yali Wang[1], Di Gao[1], Jinwang Ye[1], Xin Wang[1], Lin Fang[1], Dongqin Wu[1], Guilin Pi[1], Chengbiao Lu[2], Xinwen Zhou[1], Ying Yang*[1] & Jian-Zhi Wang*[1,3]

[1]Department of Pathophysiology, School of Basic Medicine and the Collaborative Innovation Center for Brain Science, Key Laboratory of Ministry of Education of China for Neurological Disorders, Tongji Medical College, Huazhong University of Science and Technology, Wuhan 430030, China.

[2]Department of Physiology and Neurobiology, Henan province Key Laboratory of Brain Research, Xinxiang Medical University, Xinxiang 453003, China.

[3]Co-innovation Center of Neuroregeneration, Nantong University, Nantong, 226001, China.

*Correspondence and requests for materials should be addressed to Y.Y. (email:yingyang@hust.edu.cn) and J.Z.W. (email: wangjz@mails.tjmu.edu.cn).

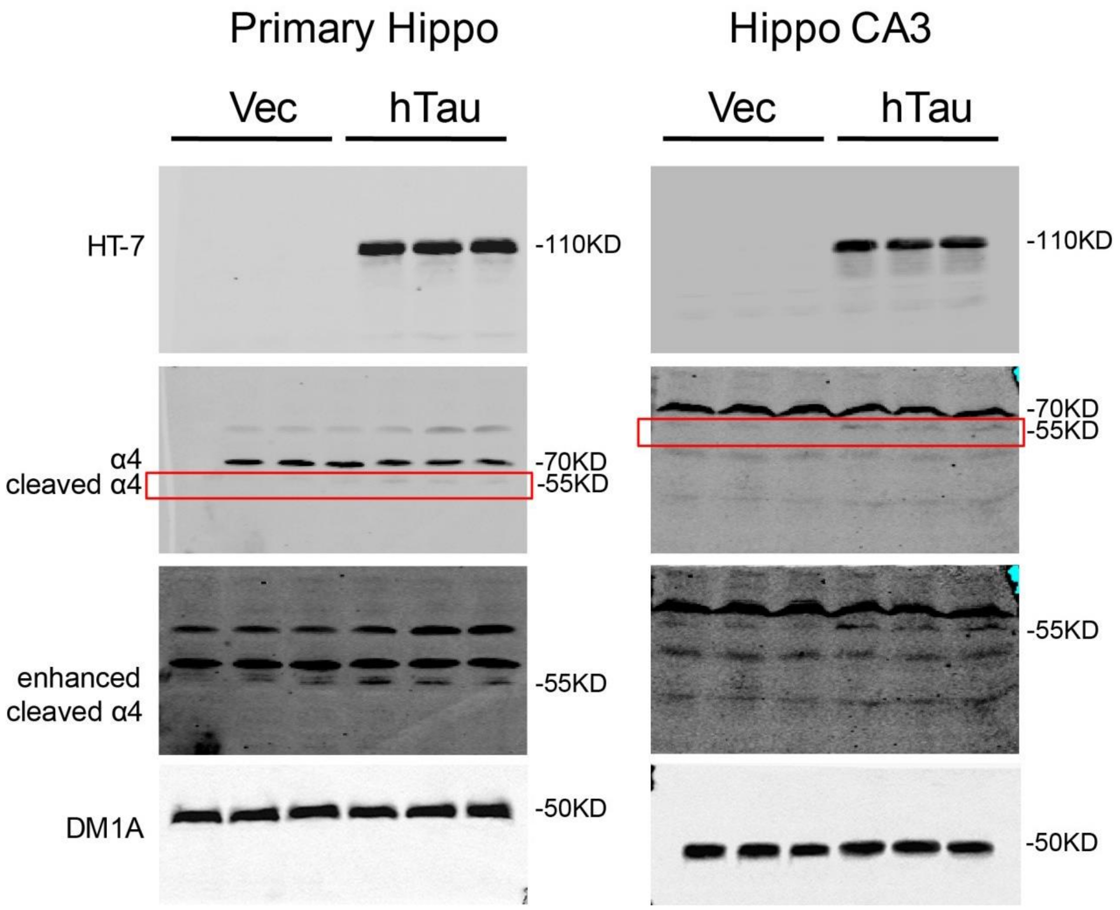

Supplementary Figure S1. Full Western blotting for Figure 1. The red dotted lines show the bands of cleaved α4 nAChRs fragment.

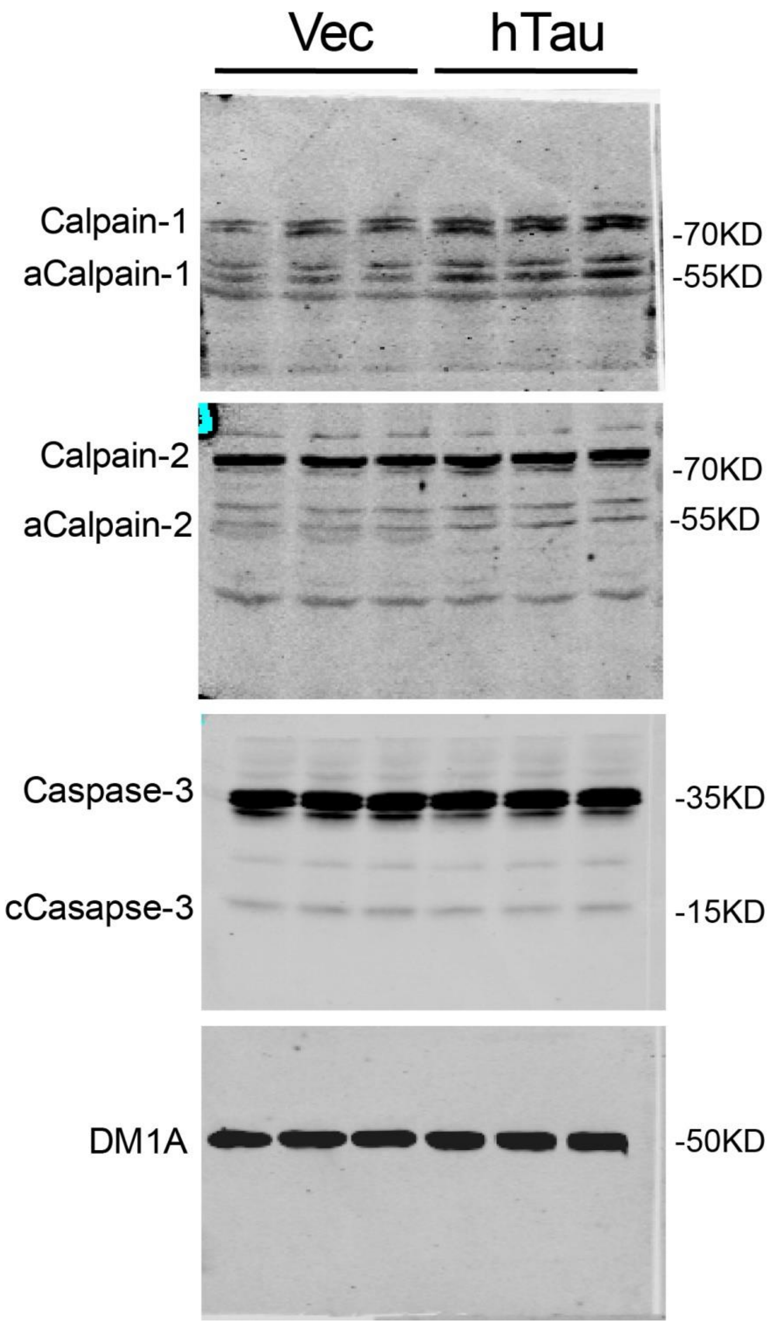

Supplementary Figure S2. Full Western blotting for Figure 3.

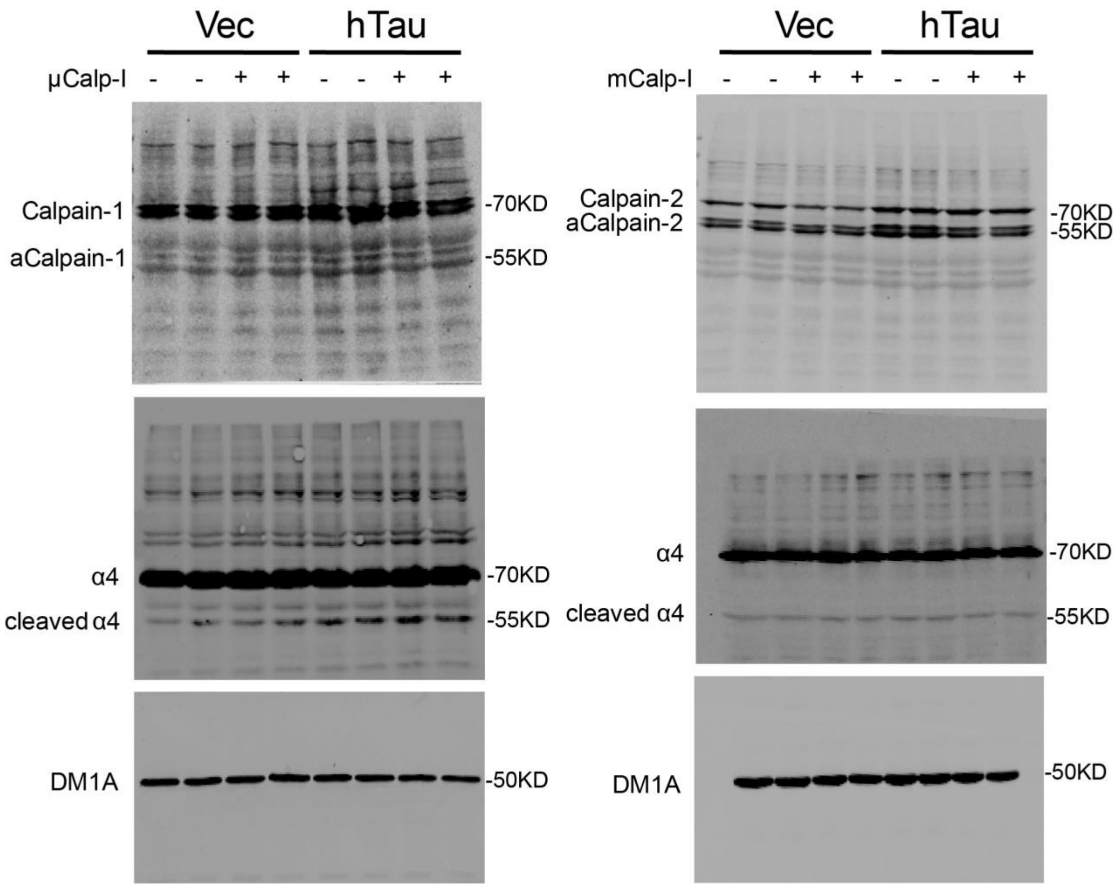

Supplementary Figure S3. Full Western blotting for Figure 4.
